# *SURF1* related Leigh syndrome: Clinical and molecular findings of 16 patients from Turkey

**DOI:** 10.1016/j.ymgmr.2020.100657

**Published:** 2020-10-23

**Authors:** Melis Kose, Ebru Canda, Mehtap Kagnici, Ayça Aykut, Ogün Adebali, Asude Durmaz, Aylin Bircan, Gulden Diniz, Cenk Eraslan, Engin Kose, Aycan Ünalp, Ünsal Yılmaz, Berk Ozyilmaz, Taha Reşid Özdemir, Tahir Atik, Sema Kalkan Uçar, Robert McFarland, Robert W. Taylor, Garry K. Brown, Mahmut Çoker, Ferda Özkınay

**Affiliations:** a Izmir Katip Çelebi University Faculty of Medicine, Department of Pediatrics, Division of Pediatric Metabolism and Nutrition, Izmir, Turkey; b Ege University Faculty of Medicine, Department of Pediatrics, Division of Nutrition and Metabolism, Izmir, Turkey; c University of Health Sciences, Antalya Training and Research Hospital, Department of Pediatrics, Division of Metabolism and Nutrition, Antalya, Turkey; d Ege University Faculty of Medicine, Department of Medical Genetics, Izmir, Turkey; e Sabanci University, Faculty of Engineering and Natural Sciences, Molecular Biology, Genetics and Bioengineering Program, Adebali Lab, Istanbul, Turkey; f Izmir Democracy University, Faculty of Medicine, Department of Pathology, İzmir, Turkey.; g Ege University Faculty of Medicine, Department of Radiology, Division of Neuroradiology, Izmir, Turkey; h Ankara University Faculty of Medicine, Department of Pediatrics, Division of Metabolism and Nutrition, Ankara, Turkey; i University of Health Sciences, Behçet Uz Children Training and Research Hospital, Department of Pediatrics, Division of Neurology, Izmir, Turkey; j University of Health Sciences Tepecik Training and Research Hospital, Department of Medical Genetics, Izmir, Turkey; k Wellcome Centre for Mitochondrial Research, Translational and Clinical Research Institute, Newcastle University, Newcastle upon Tyne, UK; l Oxford University Hospitals NHS Foundation Trust, The Churchill Hospital, Oxford Medical Genetics Laboratories, Oxford, UK

**Keywords:** COX deficiency, Leigh syndrome, Neuroregression, Next-generation sequencing, Nuclear mitochondrial disorders, *SURF1* gene

## Abstract

**Introduction:**

Pathogenic variants in *SURF1*, a nuclear-encoded gene encoding a mitochondrial chaperone involved in COX assembly, are one of the most common causes of Leigh syndrome (LS).

**Material-methods:**

Sixteen patients diagnosed to have *SURF1*-related LS between 2012 and 2020 were included in the study. Their clinical, biochemical and molecular findings were recorded. 10/16 patients were diagnosed using whole-exome sequencing (WES), 4/16 by Sanger sequencing of *SURF1*, 1/16 via targeted exome sequencing and 1/16 patient with whole-genome sequencing (WGS). The pathogenicity of *SURF1* variants was evaluated by phylogenetic studies and modelling on the 3D structure of the SURF1 protein.

**Results:**

We identified 16 patients from 14 unrelated families who were either homozygous or compound heterozygous for *SURF1* pathogenic variants. Nine different *SURF1* variants were detected The c.769G > A was the most common variant with an allelic frequency of 42.8% (12/28), c.870dupT [(p.Lys291*); (8/28 28.5%)], c.169delG [(p.Glu57Lysfs*15), (2/24; 7.1%)], c.532 T > A [(p.Tyr178Asn); (2/28, 7.1%)], c.653_654delCT [(p.Pro218Argfs*29); (4/28, 14.2%)] c.595_597delGGA [(p.Gly199del); (1/28, 3.5%)], c.751 + 1G > A (2/28, 4.1%), c.356C > T [(p.Pro119Leu); (2/28, 3.5%)] were the other detected variants. Two pathogenic variants, C.595_597delGGA and c.356C > T, were detected for the first time. The c.769 G > A variant detected in 6 patients from 5 families was evaluated in terms of phenotype-genotype correlation. There was no definite genotype – phenotype correlation.

**Conclusions:**

To date, more than 120 patients of LS with *SURF1* pathogenic variants have been reported. We shared the clinical, molecular data and natural course of 16 new SURF1 defect patients from our country. This study is the first comprehensive research from Turkey that provides information about disease-causing variants in the *SURF1* gene. The identification of common variants and phenotype of the *SURF1* gene is important for understanding SURF1 related LS.

**Synopsis:**

SURF1 gene defects are one of the most important causes of LS; patients have a homogeneous clinical and biochemical phenotype.

## 1 Introduction

Mitochondrial diseases (MD) are disorders caused by disrupted energy metabolism due to insufficiency of oxidative phosphorylation (OXPHOS) resulting from pathogenic variants in nuclear or mitochondrial DNA (mtDNA) [33]. They are individually rare, but their combined frequency is as high as 1 in 4300 and MD are considered to be the most common neurometabolic diseases of childhood [30]. The most important feature distinguishing MD from other neurometabolic diseases is undoubtedly the genomic inheritance of mitochondrial genetic material as mitochondria possess their genome. Currently, more than 350 genes of both mtDNA and nuclear origin are known to cause MD ([14]; J. [22]). Bigenomic inheritance of MD, when combined with the heterogeneous spectrum and poorly understood mitochondrial pathophysiology, causes challenges in the diagnostic process. Next-generation sequencing (NGS) technologies have revolutionized the diagnosis of MD, with the inclusion of gene agnostic whole-exome sequencing (WES) in a “genetics first” approach, increasing diagnostic yields to >60% [38].

One of the most common phenotypes of MD is Leigh Syndrome (LS, OMIM 256000), with an approximate prevalence of 1 in 40,000 live births [10]. It usually presents with failure to thrive and neurodevelopmental regression starting before 2 years of age often after an initial period of relatively normal development. Although central nervous system findings are prominent, multisystem involvement may also be seen (S. [23]). LS is genetically heterogeneous with more than 80 genetic loci reported; 30% of cases are due to pathogenic mtDNA variants, the remainder due to bi-allelic variants in nuclear-encoded mitochondrial genes(J. S. [12]).

Cytochrome *c* oxidase (COX, complex IV, OMIM 220110) deficiency is considered to be one of the most common causes of autosomal recessive LS. COX comprises 14 subunits, of which 11 are encoded by the nuclear genome and the remaining 3 by mtDNA. Besides, several nuclear-encoded chaperone proteins, including SURF1, SCO1, SCO2, COX10 and COX15, are required to assemble the holoenzyme [24,26]. Due to the vital importance of SURF1 in the human COX assembly, human SURF1 gene defects result in the failure of active COX formation and accumulation of COX assembly intermediates in tissues. Pathogenic *SURF1* variants usually present with Leigh Syndrome (LS) and are the most common cause of COX deficiency-associated LS (I.-C. [11,13]). SURF1 is necessary for correct COX assembly and a defect of this assembly factor was the first example of an indirect effect causing LS [27,35]. *SURF1* is located on chromosome 9p34 in a cluster of genes referred to as the “surfeit” genes. It encodes SURF1, which is a 300 amino acid long mitochondrial protein located in the mitochondrial inner membrane. Although *SURF1* is necessary for the assembly and maintenance of COX, its exact function is still not fully understood [17].

To date, more than 120 discrete *SURF1* gene variants have been identified in HGMD (http://www.hgmd.cf.ac.uk/ac/gene.php?gene=SURF1). However, the spectrum of *SURF1*-related MD in Turkey remains unknown. Here we present clinical, radiological, molecular and biochemical findings of 16 new patients diagnosed to have pathogenic *SURF1* gene defects.

## 2 Materials and methods

### 2.1 Individuals

We recruited 16 patients (from 14 families) who were clinically diagnosed as “SURF1-related LS” between 2012 and 2020. LS was diagnosed based on the findings of progressive neurodegeneration with evidence of brainstem and/or basal ganglia involvement, motor and mental retardation, high levels of serum / CSF Lactate, low oxidative phosphorylation or PDH activity and defects detected in the *SURF1* gene [2,9]. Patients included in this report were evaluated retrospectively and scored according to the Nijmegen Mitochondrial Disease Criteria Scale (Score 1: mitochondrial disorder unlikely; score 2 to 4: possible mitochondrial disorder; score 5 to 7: probable mitochondrial disorder; score 8 to 12: definite mitochondrial disorder) [15]. Symptom onset age, duration of follow-up, baseline symptoms, clinical and molecular findings and muscle biopsy histochemistry data were recorded. All brain MRI findings were evaluated and classified by a single consultant neuroradiologist.

The study was approved by the local ethics committee and samples from the patients were obtained in accordance with the Helsinki Declaration. Written informed consent was obtained from all parents and guardians.

### 2.2 Molecular analyses

Genomic DNA was isolated from 2 ml peripheral blood mononuclear cells using the QIAamp DNA Blood Mini kit (Qiagen, Hilden, Germany), as per the manufacturer's instructions. We measured DNA concentration with The Qubit dsDNA HS (High Sensitivity) Assay Kit (Thermo Fisher Scientific, Inc., Waltham, MA, USA). Whole exome sequencing (WES) and SURF1 sequence analysis were performed.

#### 2.2.1 Whole exome sequencing

Whole exome sequencing was performed using an Ion S5 ™ Sequencer. Ion AmpliSeq Exome RDY Kit was used according to the manufacturer's protocol. Ion reporter software was used to analyze pathogenic variants. All variants were assessed individually according to the clinical phenotype, MAF (minor allele frequency) score and pathogenicity scores calculated by prediction programs. Variants were filtered to retain nonsynonymous changes with a minor allele frequency (MAF) of <0.01 using combined datasets from the 1000 Genomes Project, the Exome Variant Server project, and Genome Aggregation Database (gnomAD). The potential functional impacts of the disease candidate variants were assessed using SIFT (http://sift.jcvi.org/) and PolyPhen-2 (http://genetics.bwh.harvard.edu/pph2/), MutationTaster (http: //www.mutationtaster.org/) and VarSome. All genetic variants were screened by pathogenicity, mode of inheritance and clinical phenotypes. Finally, candidate pathogenic variants identified by WES were verified with Sanger sequencing.

#### 2.2.2 *SURF1* sequence analysis

All coding *SURF1* (NM_003172.3) exons and their flanking regions were amplified by PCR. The amplicons were cleaned up using Sephadex (GE Healthcare) before sequencing on an ABI PRISM 3130 DNA analyzer (Life Technologies, CA, USA) using Big Dye Terminator Cycle Sequencing V3.1 Ready Reaction Kits (Life Technologies, CA, USA).

#### 2.2.3 Validation by Sanger sequencing

Sanger sequencing of the genomic variants identified by exome sequencing or targeted gene sequencing was performed for all patients and their families. Sanger sequencing was used to validate the pathogenic variants within families on 3500 genetic analyzer (Applied Biosystems, Foster City, USA). The sequencing results were analyzed using CLC genomic workbench software. For the clinical interpretation of variants, allele frequency data were obtained from various databases, including gnomAD (http://gnomad.broadinstitute.org/) and ExAc (http://exac.broadinstitute.org/). The pathogenicity of variants was assessed using in silico prediction tools, such as PolyPhen-2 (http://genetics.bwh.harvard.edu/pph2), SIFT (http://sift.jcvi.org), and MutationTaster (http: // www. mutationtaster.org) and Human Splicing Foundation (http://www.umd.be/hsf/). Variants were classified according to The American College of Medical Genetics and Genomics (ACMG) [25].

### 2.3 Nomenclature

All pathogenic variants are described according to accepted HGVS nomenclature. Nucleotide numbers are derived from complementary DNA (cDNA) sequences (GenBank accession no. *SURF1*, NM_003172.2).

### 2.4 Evolutionary studies and 3D structure model of SURF1

Human SURF1 protein (Q15526) was retrieved and queried through BLASTP against the reference proteins database [1,36]. Significant hits were compiled and aligned using MAFFT version 7.221 *E*-INS-i algorithm [8]. Maximum likelihood phylogenetic tree was built with RAxML 8.2.11 with automatic protein substitution model selection (PROTGAMMAAUTO) and 100 rapid bootstrapping parameters [29]. Gaps in human SURF1 sequence and corresponding positions in other sequences were removed. Conservation scores were calculated using the Bio3D R package based on amino acid identities [5]. Because there was no available human SURF1 protein structure, secondary structure was generated with PSIPRED and the tertiary structure was built with trRosetta programs [6,39]. Missense variants and allele frequency information of the *SURF1* gene (ENST00000371974.3 *) were gathered from gnomAD browser [7]. Protein model visualization analysis was performed UCSF Chimera [18].

### 2.5 Muscle biopsy

Muscle biopsy was performed in 11 patients. Skeletal muscle was obtained by open surgical biopsy of the gastrocnemius muscle as a diagnostic procedure. Pathological studies included light microscopic assessment of frozen sections. Samples were frozen in isopentane, cooled in liquid nitrogen, and 8- to 12-μm sections were cut in a cryostat. Slides were stained with hematoxylin-eosin, Masson's trichrome, modified Gomori's trichrome (Engel-Cunningham modification), oil red-O, Periodic Acid Schiff (PAS), and PAS with diastases stains. For enzyme histochemistry, Nicotinamide Adenine Dinucleotide tetrazolium reductase (NADH-TR), succinate dehydrogenase (SDH), cytochrome *c* oxidase (COX) and sequential COX-SDH reactions were undertaken [3,4].

### 2.6 Statistics

All analyses were performed by using the SPSS 22.0 statistical package program. Frequency and percentage values were used to describe classifier variables, and mean and standard deviation or median and minimum-maximum values were used to describe continuous variables. A chi-square test was used to identify for the relationship between classifier variables and an independent samples *t*-test was used to compare the mean scores of the two groups. The study was performed at a 95% confidence level (*p* < 0.05 was considered statistically significant).

## 3 Results

### 3.1 Demography

The median age of 16 patients (8 male, 8 female) from 14 pedigrees was 60 months years (range: 0–216 months). Parental consanguinity was reported in 10 of the 14 families. Nine patients were Turkish, 4 Iranian and 3 Syrian.

### 3.2 Clinical features

#### 3.2.1 Presenting symptoms

Median age at onset was evaluated as 6 months (range: 0–24 months). Three patients had symptoms with the neonatal onset and all died within the first 6 months of life. In two of these patients, MD was investigated in the neonatal intensive care [P6, P12] and they were monitored because of apnoeic spells which began in the first 48 h of life. Lactate levels increased following discontinuation of mechanical ventilation. The third patient with neonatal-onset symptoms had a congenital diaphragmatic hernia [P3]. This patient was operated on twice in the neonatal period and again at 6 weeks old. She died at the age of 4 months.

Initial symptoms appeared within the first year and in the second year of life in 10 (62.5%) and 6 (37.5%) patients respectively. The most common initial symptoms were developmental delay (6/16; 37.5%), nutritional difficulty (6/16 37.5%) and episodic decompensation (5/16; 31.25%). Subsequent features included neurodegeneration (4/16; 25%), hypotonicity (3/16; 18.7%), growth failure (3/16; 18.7%), ataxia (3/16; 18.7%), seizure (1/16; 6.2%) and dystonia (1/16; 6.2%). At least two or more initial symptoms were observed in 10 patients (62.5%) (Fig. 3A, Table 1).

**Table 1:**
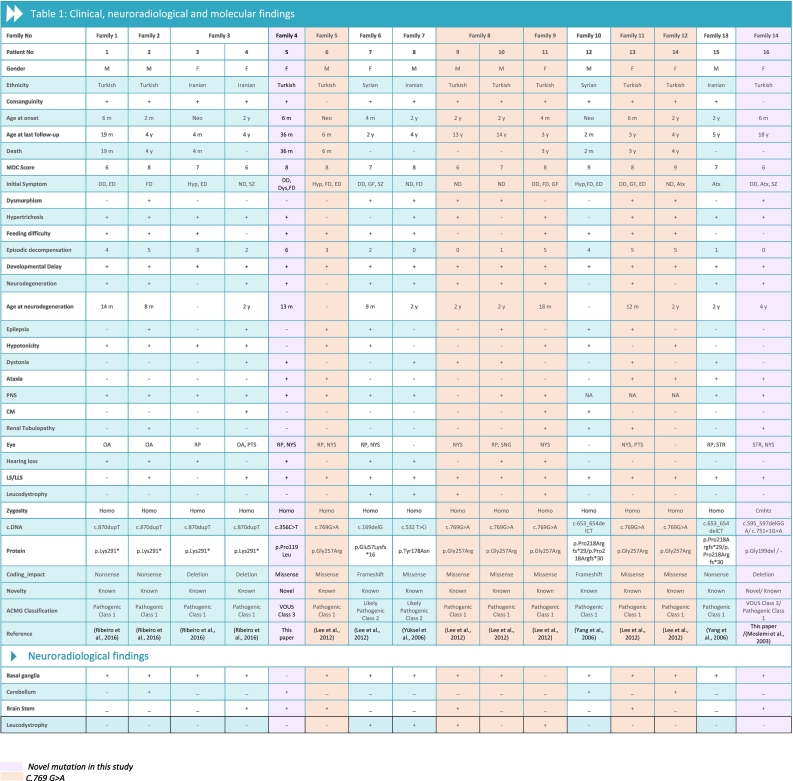
Clinical, neuroradiological and molecular findings.

CM: Cardiomyopathy, LS: Leigh syndrome, LLS: leigh-like syndrome, homo: homozygous, cmhtz: compound heterozygous, Neo: Neonatal, Atx: Ataxia, Dys: Dystonia, ED: Episodic decompensation, Hyp: Hypotonicity, FD: Feeding difficulty, GF: Growth failure, ND: Neurodegeneration, NA: Not available, RP: Retinitis pigmentosa, OA: Optic atrophy, SNG: Supranuclear gaze palsy, PTS: Pitosis, NYS: Nystagmus, STR: Strabismus, SZ: Seizure, SURF1 reference sequence (NM_003172.2).

#### 3.2.2 Neurological findings and neuroimaging

Among 16 patients, 14 (87.5%) were found to meet LS or Leigh-like syndrome (LLS) criteria. The MDC score ranged from 6 to 9 points (mean: 7.8 ± 0.9). The most common neurological signs were developmental delay (15/16; 93.7%), neurological regression (12/16; 75%). The average age of neuroregression symptoms was 13.5 months (range: 8–24 months). All patients underwent MR imaging. The scans showed bilateral basal ganglion and brainstem involvement, leukodystrophy findings and cerebellar involvement. As a new finding, distinct from previously published reports, one patient [P6] had isolated middle cerebellar peduncle involvement (Table 1).

#### 3.2.3 Other clinical features

Hypertrichosis was detected in 12 (75%) patients (Fig. 3). Nutritional difficulties were detected in 11 (68.7%) patients. Eight (50%) patients had dysmorphic findings. Renal tubulopathy and cardiomyopathy were observed in 5 (31.2%), and 3 (18.7%) patients respectively (Table 1).

#### 3.2.4 Biochemical findings

The most common laboratory abnormality was found to be mild elevation of blood lactate (15/16; 93.7%). This was followed by metabolic acidosis in 9 (56.2%) patients, respiratory alkalosis in 4 (25%), and hyponatremia in 3 (18.7%) due to renal tubulopathy. The patients' average minimum serum lactate values was 3.1 ± 0.99 mmol/l (median: 3.04, range: 2–4.8), the average maximum serum lactate values was 5.5 ± 1.8 mmol/l (median: 5.68 range: 3.45–8.36) and the cerebrospinal fluid (CSF) lactate level was 4.2 mmol/l (range: 3.09–7) (Fig. 3B, Table 2).

**Table 2:** Biochemical and Muscle Histochemical Findings.

Patient no	Cerebrospinal fluid Lactate (mg/dl)	Minimum serum lactate (mg/dl)	Maximum serum lactate (mg/dl)	Red ragged fiber	Muscle fiber size changes	COX Histochemical reaction	Neutral lipid accumulation
1	42	41	92	−	Type 1	R	+
2	51	36	78	−	Type 1	R	+
3	NA	31	72	−	Type 1	Abs	+
4	NA	24	41	NA	NA	NA	NA
5	42	36	68	NA	NA	NA	NA
6	41	51	82		−	Abs	+
7	33	26	46	+	−	Abs	+
8	NA	28	41	−	−	Abs	−
9	NA	31	39	NA	NA	NA	NA
10	39	22	42	−	Type 1	R	+
11	NA	42	57	NA	NA	NA	NA
12	44	53	81	−	Type 1	Abs	+
13	NA	41	77	NA	NA	NA	NA
14	NA	44	88	−	Type 1	R	+
15	31	25	39	−	Type 1	R	+
16	NA	22	38	−	Type 1	Abs	+

Abs: Absent, R: Reduced, COX: cytochrome c oxidase, N.A.: Not available.

#### 3.2.5 Muscle histochemistry

Muscle biopsy was performed in 11 of 16 patients. Lipid content was increased in 10/11 patients (%91). Type 1 predominance was noted in 8/11 patients (72%). COX histochemistry demonstrated the absence of COX reactivity in 6/11 (54.5%) and a global decrease in COX reactivity in 5 patients 5/11 (45.4%). RRF were noted in only one patient (9%) (Table 2).

#### 3.2.6 Molecular findings

Nine different variants were detected on 28 alleles from 14 unrelated families. Of the 9 variants; 3/9 (33.3%) were missense, 3/9 (33.3%) were nonsense, 2/9 (22.2%) were deletion; 1/9 (11.1%) were frameshift variants. The same variants were detected on both alleles in 15/16 patients; the remaining proband was compound heterozygous for *SURF1* variants. The c.769G > A was the most common variant with an allelic frequency of 42.8% (12/28), c.870dupT [(p.Lys291*); (8/28 28.5%)], c.169delG [(p.Glu57Lysfs*15), (2/24; 7.1%)], c.532 T > A [(p.Tyr178Asn); (2/28, 7.1%)], c.653_654delCT [(p.Pro218Argfs*29); (4/28, 14.2%)] c.595_597delGGA [(p.Gly199del); (1/28, 3.5%)], c.751 + 1G > A (2/28, 4.1%), c.356C > T [(p.Pro119Leu); (2/28, 3.5%)]were the other detected variants. The c.595_597delGGA (p.Gly199del) and c.356C > T (p.Pro119Leu) variants appeared to be novel in our patient cohort. Ten (62.5%) patients were molecularly diagnosed using whole exome sequencing (WES) approach, 4 (25%) with targeted *SURF1* sequencing, 1 (6.25%) with a targeted exome approach and 1/16 (6.25%) with whole genome sequencing (WGS) (Table 1).

#### 3.2.7 Modelling the *SURF1* variants and evolutionary studies

**Fig. 1:**
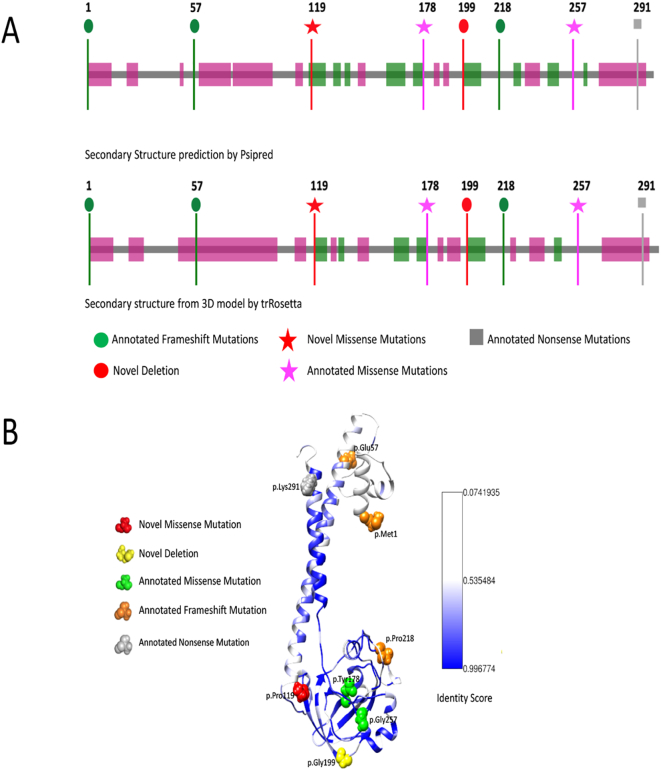
: SURF1 structure prediction. (A) Secondary structure of human SURF1 predicted by PSIPRED at top, and below secondary structure corresponds to tertiary structure generated by trRosetta are shown. Pink and green regions are predicted to be alpha-helices and beta-sheets, respectively. The uncolored gray regions correspond to the disordered regions. Pinpointed mutations reported in this study are colored and shaped based on their mutation types and novelty. (B) The tertiary structure of SURF1 built with trRosetta. Novel missense mutation p.119 is shown as red spheres, and previously annotated missense mutations p.178, p.256 are shown as green spheres. Annotated frameshift mutations p.1, p.57, p.218, and novel deletion p.199 are shown as orange.

The tertiary structure of the human SURF1 protein is not yet available, so to gain insight into the effects of SURF1 variants on the protein, we predicted the secondary and 3D structure SURF1. The secondary structure was predicted with PSIPRED (Fig. 1A). 3D structure model of SURF1 was predicted via homology modelling using trRosetta (Fig. 1B). 3D model of the protein has a similar secondary structure with the secondary structure prediction by PSIPRED [6]. Novel p.Pro119Leu and p.Gly199del variants were found to be within the conserved SURF1 domain (Fig. 1A). The structural model of the protein suggests two long helices (N and C-terminus) and a globular alpha-beta fold in between. The globular part was shown to form a conserved fold based on the sequence analysis performed by calculating identity-based conservation scores for each amino acid position in the multiple sequence alignment (see methods).

Conservation score for the positions with the allele for the missense variants obtained from gnomAD [7] was calculated and plotted with their allele frequencies. The variants with allele frequency rare than 10^−4^ of the population were filtered out. As the allele frequency gets smaller, conservation score of an amino acid in a position gets higher, suggesting conservation as proximity of predicting the pathogenicity of an associated position. The conservation patterns and thus pathogenicity depends on the region of the position in the protein. Some parts in the protein are more conserved than others. The region that is annotated with SURF1 Pfam domain is specifically conserved relative to the other regions; 6 of 8 variants are within the SURF1 domain region.

**Fig. 2:**
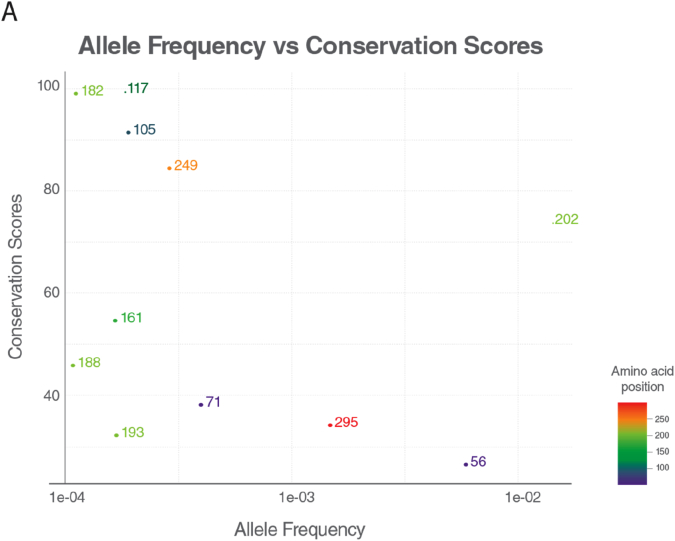

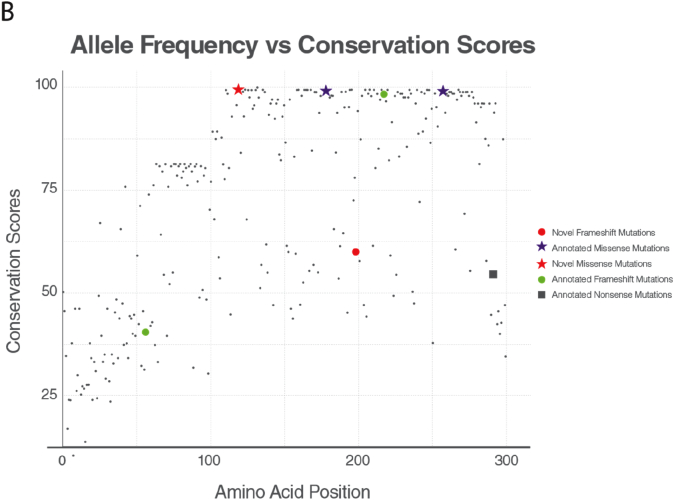

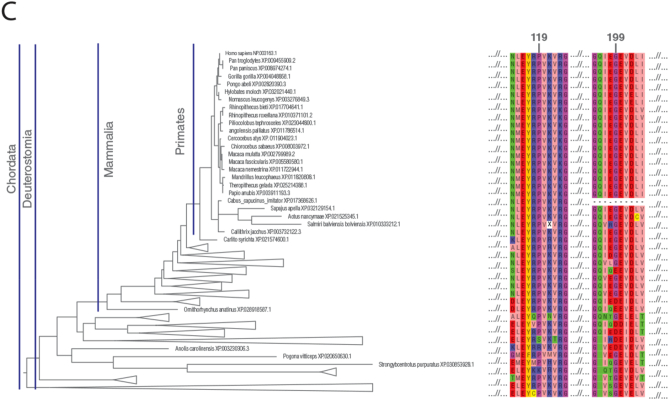
Conservation of the frequent alleles and phenotype-associated positions through phylogenetic analysis. (A) Allele frequency of observed population variants in human Surf1 protein. The variants that are more frequent than 0.1% in the population are reported only. (B) Conservation of each position in mammals. The positions with observed mutations are highlighted with the consistent color and shape scheme in Fig. 1A. (C) Maximum likelihood tree built with RAxML is shown along with multiple sequence alignment. The local multiple sequence alignment of the positions for the novel missense and frameshift mutations are plotted. Some clades are collapsed for visual purposes, the full tree and multiple sequence alignment is given in the supplementary figures.

Conservation scores were analyzed according to the phylogenetic tree (Fig. 2C). Proline residue (P119) was conserved among species (fully conserved in mammalian Fig. 2B) except in two clades p.Pro119Ser and p.Pro119Arg substitutions were observed in the phylogenetic tree. p.Pro119Ser substitution was observed only in Archelosauria (taxon:1329799). p.Pro119Arg substitution was seen only in Percomorphaceae (taxon:1489872, bony fish). No single Pro119Leu substitution was observed across all species. The fact that leucine has never been observed throughout the evolution of SURF1 and full conservation of proline at position 119 for mammals suggest that Pro119Leu substitution is likely to be pathogenic. c.595_597delGGA variant has been evaluated as a novel deletion and classified as “variant of unknown significance (VUS)” in accordance with ACMG recommendations. However; it has not been found in Genome Aggregation Database (gnomAD genomes and gnomAD exomes; mean coverage is 32.7 and 73.6 respectively). It was predicted that as deleterious based on GERP and has no any benign prediction. p.Gly199 was fully conserved among clades closer to the human (Eutheria, placentals, taxon:9347) and as far as we know, no deletions have been reported in this region to date.

#### 3.2.8 Follow-up and survival

We had 7 patients who were still alive at the time of writing the report (aged between 2y-18y). The age at death of the 9 patients (9 56.2%) ranged from 1 month to 5 years (median: 36 months). Since 2 (12.5%) patients died at home the cause of death in these 2 patients were unknown. Seven (77%) patients died from respiratory failure caused by lung infection. It was observed that 5 (71.4%) of the 7 survivors had symptom onset at 2 years and older. In our study, no difference in survival rates was observed between patients with the most common c.769 G > A (p.Gly257Arg) variant (6/16) and patients with other variants. There was no relationship between neuroregression time and time of death (p: 0.844, pearson correlation: −0.105). During the follow-up, the number of hospitalizations of patients varied between 2 and 12, and the total duration of hospitalization varied between 26 days and 8.3 months. It has been observed that the most common reason for hospitalization is episodes of infection-induced decompensation. The median number of episodes of decompensation was 3 (mean: 2.8 ± 2.0, range: 0–6). There was a statistically significant difference in the number of decompensation episodes between deceased patients and survivors with a mean number of 4.6 ± 0.9, and 1.1 ± 1.1 respectively (p: 0.001). The average minimum and maximum lactate levels were also significantly different between these two groups (Table 2). The effects of age at onset, neurodegeneration time and clinical findings on survival were evaluated. Hypotonicity (life expectancy without hypotonicity: 164.5 ± 30.7 months, with hypotonicity 37.0 ± 8.2 months; p:0.035) and feeding difficulty (median 36, range: 2–60 months, p: 0.03) were found to have significant negative effects on survival. (Fig. 5A and 5B)

## 4 Discussion

SURF1 deficiency is one of the most common causes of LS. Unfortunately, the incidence of *SURF1*-related LS in our country is currently undetermined. This work represents the first case series on SURF1 deficiency and LS from Turkey, reporting 16 new patients and 2 previously-unreported genetic variants.

### 4.1 Clinical aspects

When the clinical features of the whole population of our study are analyzed, a clinical picture that starts with developmental delay and nutritional difficulties and progresses with neurodegeneration can be observed. The most common clinical finding was a developmental delay (93%), followed by neurodegeneration and hypertrichosis (75%). While only 4 (25%) patients had neuroregression during initial diagnosis this rate increased to 75% during follow-up. Neuroregression appeared around the age of 12–13 months. Epilepsy, as another neurological finding, was observed in 43.7% (7/16) of our patients. Epilepsy has not been reported as a common symptom in the *SURF1* gene defect. The higher epilepsy frequency in our study could be explained by different genetic modifiers in different populations. Among nuclear MDs, the *SURF1* defect has been reported to be the most frequent molecular pathology associated with movement disorders. However, it has not been specified for a particular variant, approximately 52% of patients with SURF1 defects show movement disorders [37]. It has been shown that movement disorder is more prominent in patients with a missense variant and older age [2,19]. As the movement disorders, dystonia and ataxia were seen in our patients. 7 of 9 patients with movement disorders in our patient group had a missense variant. Although tremor has been frequently reported to be associated with SURF1 defects, no patient in our study presented with tremor.

Because of the broad heterogeneity of MD, specific phenotypic findings that make the diagnosis easier have been intensively investigated. Unfortunately, no strong external phenotypic features that distinguish the causes of MD have been identified. In this regard, SURF1-related MD has a slightly more advantageous position due to the high frequency of hypertrichosis. No other gene defect causing MD has such a strong link with hypertrichosis. Supporting this observation, hypertrichosis was present in 75% (12/16) of our patients. Wedatileke et al. and Lee et al. have reported hypertrichosis in 41% and 19% of their patients with SURF1 defects, respectively (I.-C. [11,37]). Although there is no definite report about the character and distribution of hypertrichosis, it has been stated that it involves predominantly the forehead and extremities [16]. In our patient group, hypertrichosis was concentrated on the back, extremities and nape of the neck causing a low hairline appearance. The presentation of hypertrichosis before the initial symptoms started has been considered as an important diagnostic clue by clinicians.

**Fig. 3:**
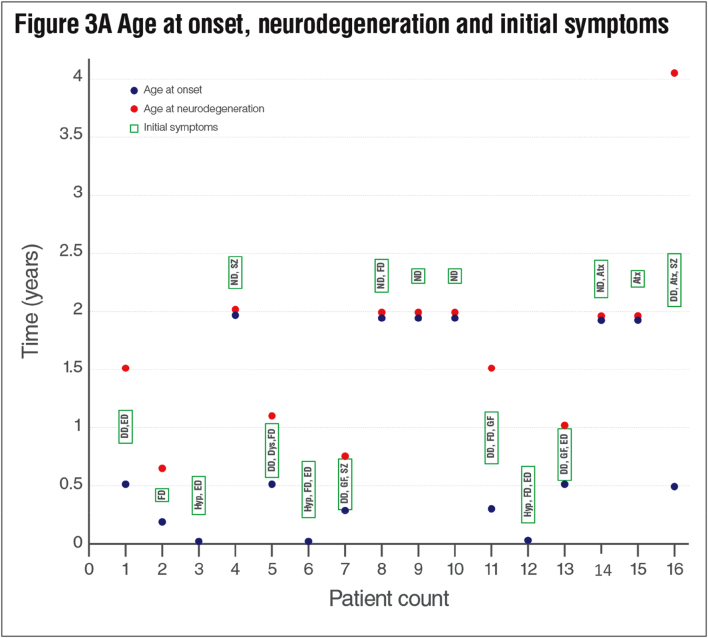

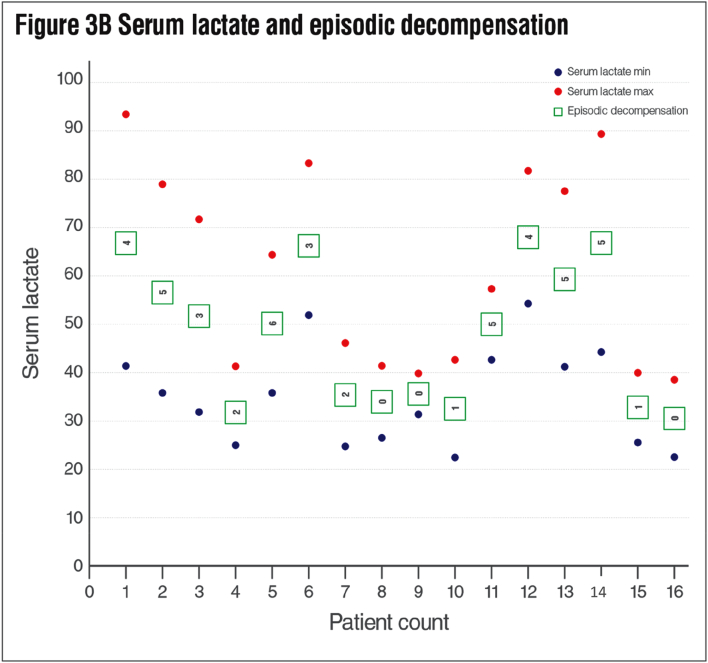
A Age at onset, neurodegeneration and initial symptoms of the patients. *ED: Episodic decompensation, FD: Feeding difficulty, Hyp: Hypotonicity, ND: Neurodegeneration, SZ: Seizure, Dys: Dystonia, GF: Growth failure, Atx: Ataxia, DD; Developmental delay.* B Serum lactate and episodic decompensation attacks of the patients.

**Fig. 4:**
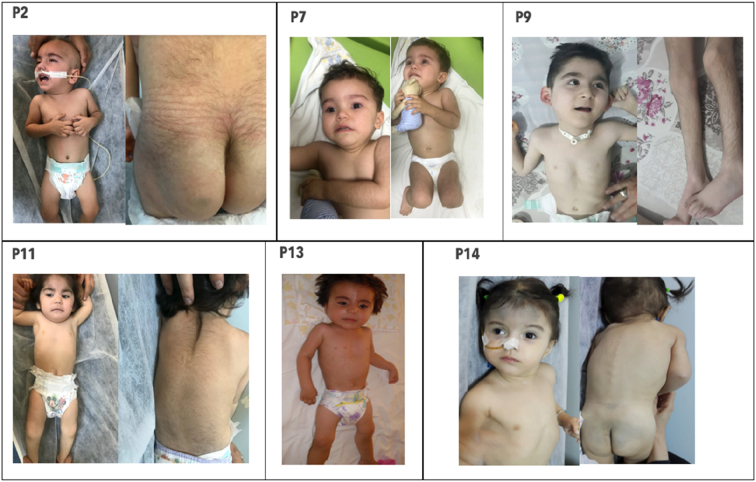
Phenotypic Features of Patients. P2: Global hypertrichosis concentrated in back, large mongolian spot on gluteus P7: Microcephaly, narrow forehead,low-set ears, hypertelorism, depressed nasal bridge, short and bulbous nose, anteverted nostrils, smooth-broad philtrum, thin lips, down-thurned mouth, hypretrichosis on extremities P9: Narrow forehead, prominent eyebrows, downslanted palpebral fissures, low-set ears, smooth-broad philtrum, thin lips, micrognathia, pectus excavatum, hypertrichosis. P11: Narrow forehead, prominent eyebrows, low-set ears, thin lips, down-thurned mouth, low hair line, hypertrichosis concentrated on nape of head and back. P13: Narrow forehead, depressed nasal bridge, short-bulbous nose, thin lips, broad chest. P14: Narrow forehead, hypertelorism, depressed nasal bridge, thin lips,large mongolian spots on forehead and gluteus.

Another important finding in terms of external phenotypic features is dysmorphic features. Numerous studies have addressed multisystemic findings secondary to the mitochondrial energy deficit caused by the *SURF1* gene defect. However, dysmorphic findings have not been emphasized strongly. Microcephaly, coarse face, narrow forehead, brachycephaly, prominent eyebrow structure, low-set ear, telecanthus, hypertelorism, microretrognathia, large mongolian blue spots and various external phenotypic findings were detected in our cases. Dysmorphic findings have been previously reported in a small number of studies. Sonam et al. described triangular face and low-set ear similar to our patients [28,31,32,40]. Although dysmorphological findings in such a large group have not been described so far, we have not observed the same prototype in all our patients. However; coexistence of microcephaly, narrow forehead and low-set ear in 5 patients has been considered remarkable (Fig. 3). (See Fig. 4.)

**Fig. 5:**
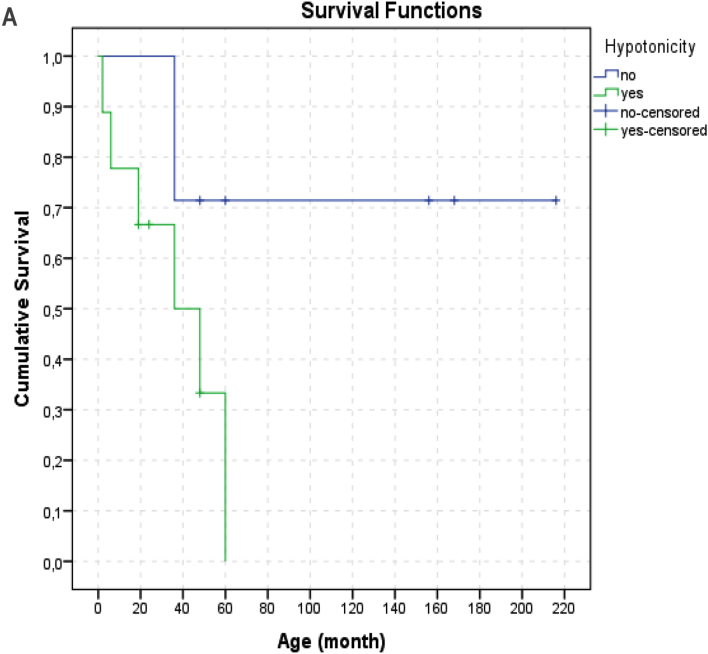

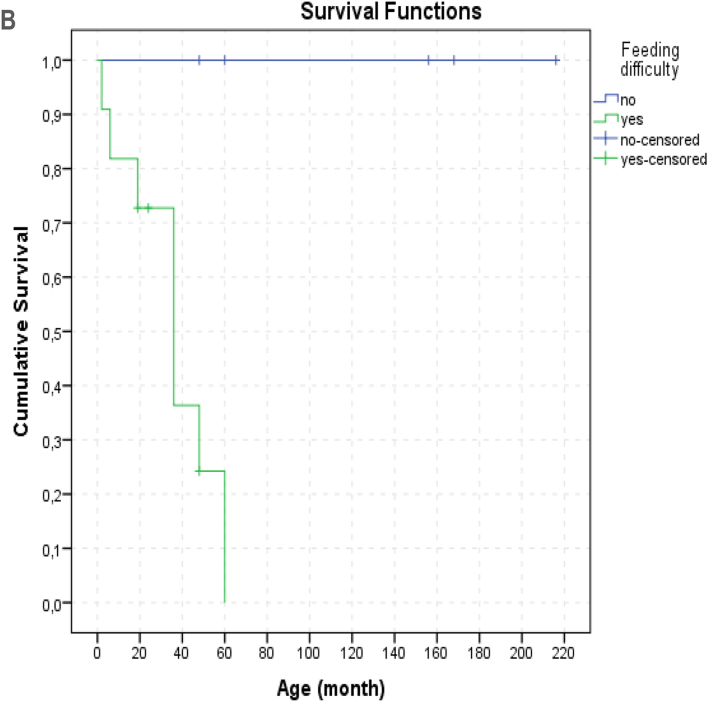
A. Kaplan-Meier survival (hypotonicity). B. Kaplan-Meier survival (feeding difficulty).

There have been some studies which report that subthalamic nucleus involvement is common and one of the specific MRI findings in patients with SURF1 defects [2]. However, in our study, only 2 patients showed this finding. Therefore it has been considered that subthalamic nucleus involvement may be less frequent than reported previously. Additionally, isolated cerebellar pedicle involvement was observed in one of our patients (Patient 6). Cerebellar pedicle involvement with brainstem involvement has been demonstrated previously in patients with SURF1 defects. However, in our study, isolated and clearly defined middle cerebellar pedicle involvement was demonstrated for the first time. It was remarkable that this patient also had newly identified pathogenic variant.

### 4.2 Biochemical and histochemical findings

Serum and CSF lactate levels in our cohort were found to be compatible with previous reports in the literature [19,37]. We also observed that the minimum and maximum serum lactate values during decompensation attacks were higher than previously reported. In our study, muscle histochemistry showed a uniform or global loss of COX activity. In our study, although all of our patients undergoing muscle biopsy revealed decreased activity, COX histochemistry may be normal in some patients with SURF1 deficiency. Wedatileke and colleagues postulate that this may be since many centres use a long incubation period in the COX reaction, an endpoint histochemical assay [37]. In our study, red ragged fibres (RRF) was seen in 1/11 (9%) patients. RRFs are more likely to occur in mitochondrial DNA-associated MD, with the presence of RRFs being an extremely rare occurrence in muscle from patients with *SURF1* gene defects [21]. It has previously been reported that RRFs were observed in two cases previously presented by Tay and colleagues [20,32].

### 4.3 Molecular diagnosis and evolutionary studies

We describe two novel *SURF1* variants (c.595_597delGGA [p.Gly199del] and c.356C > T [p.Pro119Leu]) in addition to the variants found in the *SURF1* gene to date. The c.356C > T (p.Pro119Leu) is a missense variant located in exon 5 Patient with this variant showed atypical LS with the findings of resistant apnea, dystonia, nystagmus, proximal renal tubular acidosis, middle cerebellar peduncle involvement detected on MRI. c.595_597delGGA (p.Gly199del) variant is a variant that causes the loss of amino acid Gly in the 3rd codon of the 7th exon. Ataxia, moderate mental retardation, nystagmus, proximal renal tubular acidosis were the cardinal clinical findings of the patient with this variant. Histochemical analysis of muscle biopsy demonstrated a complete absence of COX activity in this patient. Unfortunately, sufficient biopsy material could not be obtained for the respiratory chain enzyme (RCE) activity and RCE activity analysis. This variant has not been previously defined and reported to be a variant of unknown significance (VOUS) in American College of Medical Genetics Interpretation Guideline. However, the variant has not been found in GnomAD exomes and GnomAD genomes databases. To examine the pathogenicity of the variants in more detail; the 3D structure of the SURF1 protein was created (trRosetta) (Fig. 1B) and evolutionary studies were conducted. Proline residue (P119) was conserved among species (fully conserved in mammalian Fig. 2B) except in two clades p.Pro119Ser and p.Pro119Arg substitutions were observed in the phylogenetic tree. No single Pro119Leu substitution was observed across all species. The fact that leucine has never been observed throughout the evolution of SURF1 and full conservation of proline at position 119 for mammals suggest that Pro119Leu substitution is likely to be pathogenic. On the other hand, p.Gly199Glu was 51% identical among the species. We observed p.Gly199Glu, p.Gly199Gln and p.Gly199Asp substitutions, however, p.Gly199 was fully conserved among clades closer to the human. In the light of these data, it was compatible with the patient's clinical findings and histochemistry of muscle biopsy showed complete COX absence. Therefore, it has been suggested that this variant is pathogenic.

With the widespread use of NGS technologies, many improvements have been made in understanding the aetiology of MDs aetiology and elucidation of pathogenesis (J. S. [12,33,34]). In our study, a significant proportion of our patients (75%) were diagnosed using NGS technologies; the high number of patients receiving a diagnosis is also attributed to the first-line choice of WES in patients with suspected MD.

SURF1-related LS is pan-ethnic. However, the founder effect has been reported for some variants. The c.604 G > C variant was first described in China and has been shown only in Chinese patients to date [2,15,37]. C.311_312insATdel10 variant is more common in white Europeans, while c.790_800delAG variant is more common in Bangladeshis [2]. In their study on Polish patients, Pronicki and colleagues found the c.845_846delCT variant in all 21 patients included in their study [21]. The c.845_846delCT and c.312_312del10insAT variants have been reported as the most common variants in European populations. It is impossible to comment on a founder effect for Turkey due to the small number of patients in our cohort. However, we detected the c.769G > A variant in 6 patients from 5 different, unrelated families (P6, P9, P10, P11, P13, P14) living in different regions of Turkey. This variant was first detected by Lee and colleagues in 2012 in a Caucasian patient (I.-C. [11]). Since we had 6 patients with the same variants, the phenotypic findings of these patients were evaluated separately. All the patients had neurodegenerative findings starting between 12 and 24 months except one (P6), whose symptoms started in the neonatal period and who expired at 6 months of age. The patients died before the age of 4 years; except for P9 and P10 who are still alive. Wedatilake Y and colleagues reported a median onset of neurodegeneration at 19 months and a median survival of 5.4 years in their patients with SURF1 defect; however, patients living longer than 10 years have also been reported [37]. Considering these data, it is not possible to differentiate the clinical prognosis of c.769 G > A from other missense *SURF1* variants.

### 4.4 Follow-up and survival

Although long-term survival is not common in SURF1 deficiency, patients over 10 years of age have also been described in the literature. In our patient group, one of the patients (P16) was 18 years old when she was first diagnosed to have SURF1 related LS and in recent communication with the family, it was learned that she was alive and 20 years old. To the best of our knowledge, she is the oldest known patient after the 22-year-old patient reported by Abramczuk et al. [19]. In our study, 3 patients (P9, P10, P16) lived longer than 10 years and are still alive. In previous studies, patients with a long life span were generally patients without neuroregression so we evaluated our patients in this respect [19,37]. However, in our study, we found that these three patients, and even all of our patients who are still alive, have neuroregression. When we look at whether there is a relationship between the start time of our patients' neuroregression and survival, we saw that the time of neuroregression did not affect survival in our patients. However, we think that this difference is due to the difference in the number of patients since 4 of our 7 living patients are still under the age of 10 and our patient group is small compared to the large cohorts. When our patient group was evaluated for survival, median survival was 5 years. In the study of Wedatileke et al., survival was reported as 5.4 years [37]. In our study, we found that there was a statistically significant difference in the minimum and maximum plasma lactate values between deceased and surviving patients. When the literature on this subject is examined, in the study of Abramczuk et al. who have the oldest patient group serum lactate levels of surviving patients are observed to be quite low.

Our study has some limitations, the first and most important limitation is that unfortunately, we could not perform sufficient tissue studies in all our patients, especially patients with novel S*URF1* variants, another limitation is that there has been no other study from our population to compare our results. Especially in terms of evaluating the frequency of the c.769G > A variant in our country, the in-house databases of the centers where large genomic data studied should be scanned.

## 5 Conclusion

We present the clinical, molecular data and natural history of 16 patients from 14 families with SURF1-related MD. Like all Mendelian disorders, SURF1 deficiency is estimated to be more common in Turkey than other countries as the frequency of consanguineous marriage is high. Our study is the first case series reported from Turkey, identifying dysmorphic findings and molecular defects in a large series of patients with SURF1 deficiency. A lack of sufficient tissue compromised further studies, but we were able to model the 3D structure of the SURF1 protein and use this to undertake advanced evolutionary studies for pathogenicity analysis. As the availability of NGS technologies increases and their costs decrease, the use of different -omic technologies will enable both the identification of SURF1 deficiency patients and the better understanding of the pathophysiology of the disease and consequently the development of treatment options.
